# Three-dimensional time-resolved trajectories from laboratory insect swarms

**DOI:** 10.1038/sdata.2019.36

**Published:** 2019-03-05

**Authors:** Michael Sinhuber, Kasper van der Vaart, Rui Ni, James G. Puckett, Douglas H. Kelley, Nicholas T. Ouellette

**Affiliations:** 1 Department of Civil and Environmental Engineering, Stanford University, Stanford, CA 94305, USA; 2 Department of Mechanical Engineering, Johns Hopkins University, Baltimore, MD 21218, USA; 3 Department of Physics, Gettysburg College, Gettysburg, PA 17325, USA; 4 Department of Mechanical Engineering, University of Rochester, Rochester, NY 14627, USA

**Keywords:** Statistical physics, thermodynamics and nonlinear dynamics, Biological physics

## Abstract

Aggregations of animals display complex and dynamic behaviour, both at the individual level and on the level of the group as a whole. Often, this behaviour is collective, so that the group exhibits properties that are distinct from those of the individuals. In insect swarms, the motion of individuals is typically convoluted, and swarms display neither net polarization nor correlation. The swarms themselves, however, remain nearly stationary and maintain their cohesion even in noisy natural environments. This behaviour stands in contrast with other forms of collective animal behaviour, such as flocking, schooling, or herding, where the motion of individuals is more coordinated, and thus swarms provide a powerful way to study the underpinnings of collective behaviour as distinct from global order. Here, we provide a data set of three-dimensional, time-resolved trajectories, including positions, velocities, and accelerations, of individual insects in laboratory insect swarms. The data can be used to study the collective as a whole as well as the dynamics and behaviour of individuals within the swarm.

## Background & Summary

In nature, many species organize in groups or aggregations that exhibit temporally and spatially complex patterns and dynamics^1^. This behaviour can be observed in bird flocks^2–4^, fish schools^5^, and insect swarms^6,7^, among others. This group behaviour often appears to be collective, so that the group as a whole has distinctly different properties from those of the individuals^7^. It is widely believed that group behaviour is beneficial. Thus, substantial work on the nature, advantages, and origins of collectivity has appeared in recent years^5,8–10^. In addition to being of fundamental biological interest, understanding collective behaviour has also emerged as an important topic in bio-inspired engineering to enable the design of distributed robotic systems that can handle tasks in robust and efficient ways^11,12^.

**Figure 1:**
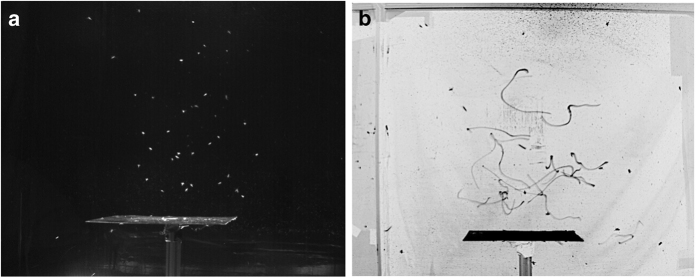
Images of midge swarms in the laboratory, taken with the same cameras and illumination used to obtain the data. (**a**) A snapshot from a swarming event. Midges are swarm above a square black plate that serves as a nucleation point for the swarm. (**b**) An inverted, long exposure of the swarm, showing the trajectories of individual midges. The contrast in both images is enhanced for better visualization.

With recent technological advances in imaging technology, the study of animal aggregations has increasingly focused on the detailed observation of individuals within the group to provide simultaneous measurement of individual and group behaviour. For larger animals such as birds or fish, the task of tracking individuals can become challenging due to visual occlusions of individuals and potential large-scale translational movement of the group^4^. Many larger animals must also be studied in the wild, which brings additional complications. In particular, it can be difficult to disentangle the effects of environmental stimuli, which can simultaneously affect many individuals, from the intrinsic collective behaviour of the group^13,14^. To remove any confounding external stimuli, we performed laboratory observations on swarms of *Chironomus riparius*, a non-biting midge species that consistently and predictably forms mating swarms over visual cues^15^ (see Fig. 1). As we have shown elsewhere, these swarms are a useful and convenient system for investigating collective behaviour^7,14,16,17^, including by allowing potentially powerful analogies to materials science^18,19^, thermodynamics^20^, and gravitating systems^21,22^.

Here, we present a dataset of such individual trajectories in laboratory insect swarms. We use a three-camera setup to reconstruct the three-dimensional positions, velocities, and accelerations of each individual midge during the swarming process. This temporally and spatially resolved data allows for statistical, dynamic, and topological analyses, and can give insights in the behaviour of both individuals and of the group as a whole.

## Methods

### Insect colony

The data described here was obtained from imaging swarms of *Chironomus riparius* midges living in a self-sustaining laboratory colony^7^. We established the colony from initial egg sacs purchased from Environmental Consulting and Testing, Inc. The midges are kept in a (122 cm)^3^ cubical enclosure made of acrylic for easy optical access. The room in which this enclosure sits is maintained at a constant 22 °C and 50% humidity, with no natural light sources. The enclosure is illuminated by an overhead light set to a circadian cycle, providing 16 h of light and 8 h of darkness per day.

Male midges spontaneously form mating swarms twice daily, at (laboratory) dusk and dawn. We typically observe larger swarms at dusk; most of the data reported here was acquired from dusk swarms. Females do not participate in the swarming behaviour, but will occasionally fly through the swarms to find mates. These events are rare and are not present in the data provided here.

The insect colony setup is similar to what was described in references^7,14,16–18,23^, though there the enclosure was smaller. The larger midge enclosure here allows for larger swarms to form that are still not influenced by the walls.

### Setup and Procedure

Swarms of *C. riparius* are well known to nucleate over visual features on the ground^15,24^. In the wild, such features may be, for example, tree stumps or stream banks. In the laboratory, we provide a 31×31 cm^2^ “swarm marker” (in our case, a black square plate) for this purpose (see Fig. 1). In addition to encouraging the formation of swarms, the marker also allows us to position swarms in the midge enclosure so that we can ensure their visibility by our imaging system and prevent them from drifting in space or interacting with the walls of the enclosure. Note that swarms do not tend to fill the entire enclosure, but rather remain far from the walls^7^. As such, the insects are not directly constrained by the size of the laboratory environment.

Static properties such as the size and shape of the marker can affect the behaviour of very small swarms, but do not play a strong role in the morphology or behaviour of swarms larger than about 10 individuals^23^. In contrast, dynamic movement of the swarm marker does affect the swarm noticeably.The data we present here was obtained using a static marker.

**Figure 2:**
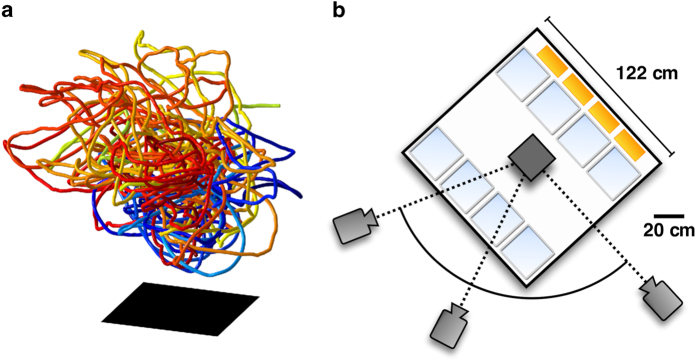
Midge trajectories and experimental setup. (**a**) Long reconstructed trajectories from a single observation. Shown are those trajectories from Ob14 that are longer than 35 s. The swarm marker is shown by a black square. (**b**) Schematic top view of the experimental setup. Swarms form inside a cubical acrylic enclosure measuring 122 cm on a side and are imaged using three cameras mounted outside the enclosure. The swarm marker is roughly positioned in the center of the enclosure. The enclosure contains eight midge development tanks (light blue) and four infrared LED arrays (yellow; additional arrays on top of the enclosure are not shown).

We image the swarms using three hardware-synchronized Point Grey Flea3 cameras, recording 8-bit greyscale images with a spatial resolution of 1280 by 1024 pixels at a rate of 100 Hz. Using an array of near-infrared LEDs, the swarms are illuminated at a wavelength that is visible to the cameras but not to the midges, so that their behaviour is not disturbed by lighting. Each swarming event is filmed for approximately 2 to 5 min, corresponding to roughly 10000 to 20000 frames of data. The cameras are arranged outside the enclosure in a horizontal plane, as sketched in Fig. 2b, with angular separations of approximately 30° and 70°. To calibrate the imaging system, we assume a standard pinhole camera model^25^. The cameras are calibrated using a target mask consisting of a regular dot pattern^26^ that is positioned in the center of the enclosure and removed before swarming begins. The conceptual design of the experiment and the data acquisition follows the description in ref.^7^, with camera locations and illumination setup adjusted to account for the larger midge enclosure.

### Data Treatment

**Figure 3:**
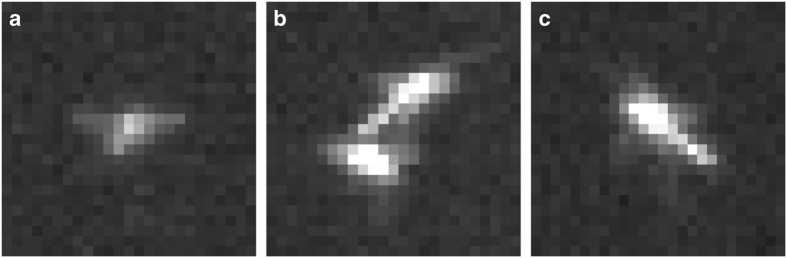
Enlarged images of midges as captured by our cameras. (**a**) A representative single midge as detected by the image processing algorithm. (**b**) A typical occurrence of a non-circular detected area that corresponds two two individual midges. These areas are split into two separate groups of pixels, each corresponding to a single midge, to improve the stereomatching rate. (**c**) An example of a large detected area that corresponds to a single midge that may potentially be obstructing another midge. These areas are duplicated in place to allow the line of sight through this midge from one camera to intersect with multiple lines of sight from the other cameras.

To track the motion of individuals in the swarm, we followed the methodology described in ref.^7^. We first located midges in each camera frame by finding the centroids of regions that had sufficient contrast with the background and were larger in area than an appropriate threshold *A*_1_, after the average of all frames was subtracted (see Fig. 3a). To improve on the detection method, centroid coordinates of circular regions above a second larger threshold *A*_2_ (see Fig. 3c) were duplicated as they potentially corresponded to two midges almost completely overlapping from the viewpoint of a single camera. This allowed the stereomatching to correctly distinguish two midges that were partially obstructed in the field of view of one camera. Highly non-circular regions above a third area threshold *A*_3_ were additionally split into two spatially separated midges because they may potentially correspond to two distinct midges that overlap only slightly in the frame (see Fig. 3b). *A*_1_ was chosen to be about 15 pixels, which for the given illumination and camera setup proved to be large enough to prevent unnecessary false positives. *A*_2_ was about 100 pixels which is larger than any typical single midge observed, and *A*_3_ was about 150 pixels to reduce the error in finding the center of split midges. Note that while for the observations in this dataset the illumination and camera setup remained constant, in general these parameters do strongly depend on the illumination level and the distance of the cameras from the swarm center.

Combining the two-dimensional positions on the frames obtained from each camera and the relative coordinates of the cameras (found using a standard calibration method based on Tsai’s model^25^), we constructed an epipolar line of sight for each midge image on each camera. Near intersections of triplets of these epipolar lines then determine the location of the midges in three-dimensional space. Here, we only considered midges that were seen by all three cameras. Although in principle two views are sufficient for stereoimaging, in practice at least three cameras are typically required to resolve ambiguities and avoid false identifications^27^. Arranging all three cameras in a horizontal plane, as we have done here, can still leave some residual ambiguity. However, this situation occurs infrequently and is more than compensated for by the simpler and superior camera calibration that can be obtained when all the cameras are positioned approximately orthogonally to the walls of the midge enclosure.

After determining the three-dimensional positions of the midges, we tracked their motion in time using a predictive tracking algorithm originally developed to study turbulent fluid flows^27^. This algorithm proceeds by using the prior flight history of a midge to estimate the expected position of the midge in future frames; the real midge that is found closest to the estimated position is linked to the trajectory^27^. We set the parameters of this algorithm conservatively, so that ambiguities in the tracking (as can be caused by, for example, midges that come very close together or midge positions that are missing or misidentified) led to trajectory segments ending rather than to tracking mistakes. Subsequently, however, we tested whether we could splice together trajectory segments by re-tracking them in a six-dimensional position-velocity space that serves to spread out the potential matches and resolve ambiguities^28^. To do this, all tracks obtained via the tracking algorithm were projected forward and backward in time using positions and velocities at the track endpoints. If the distance in position-velocity space of the forward projection of one trajetory and the backward projection of another trajectory falls below a threshold, one can assume that those tracks belong to the same individual midge, and the trajectory segments can be joined^28^.

Once the trajectories were identified, we computed velocities and accelerations by convolving the trajectories with a Gaussian smoothing and differentiating kernel^29^, thereby avoiding noise that can be introduced by simple finite differences^23^. For the data presented here, the convolution kernel was chosen to have a standard deviation of 2 frames, and the position information from 9 frames was used to calculate each derivative.

Our time resolution was sufficient to capture even the most intense acceleration events displayed by the midges^7^. We note that since midge swarms are very dilute, tracking is relatively easy for these data sets. Sample midge trajectories are shown in Fig. 2a.

### Code availability

Code for stereomatching and tracking is available from the corresponding author upon request.

## Data Records

The dataset (Data Citation 1) contains 19 individual swarming events (see Table 1), which each contain the trajectories of all the midges within the swarm. The swarm recordings were between 100 and 200 s long, and the swarms contained between 15 and 94 individuals. Each swarm measurement is stored in a .csv file. The data is organized in 11 columns (see Table 2), with each line corresponding to one individual midge at one specific time. The first column contains a unique numerical identifier *id* corresponding to a single midge. The second through fourth columns contain the *x*, *z* and *y* coordinates, respectively, of the midge in mm, with *z* pointed antiparallel to gravity. The fifth column contains the time stamp *t* of that frame in seconds. The sixth through eighth columns contain the velocities *v*_*x*_, *v*_*z*_, and *v*_*y*_ in the *x*, *z*, and *y* directions, respectively, in mm/s. The ninth through eleventh columns contain the accelerations *a*_*x*_, *a*_*z*_, and *a*_*y*_ in the *x*, *z*, and *y* direction, respectively, in mm/s^2^.

## Technical Validation

The image processing step of our method was tested by comparing the algorithmic results with those obtained by human identification on a representative sample of images. By appropriately tuning the thresholds, all midges identified by eye were automatically detected by our algorithm. The tracking algorithm used in this study has been thoroughly tested against numerical simulations of particles in turbulent flows^27^, a scenario in which individual particles display much more erratic behaviour and much stronger accelerations than the swarming midges. To do this, particle trajectories were generated by direct numerical simulation of the Navier-Stokes equations for the case of a turbulent flow. The trajectories were parameterized by time, and lists of the time-resolved positions (without any indication of which position belonged to which trajectory) were fed into the tracking algorithm. Since the trajectories of the simulated particles were known *a priori*, it was possible to evaluate the performance of the tracking algorithm quantitatively by comparing the true trajectories with the output of the algorithm^27^. In the case of low particle densities, as is the case in the midge swarms, tracking mistakes were negligible. We did not directly assess the performance of our tracking algorithm on simulated midge trajectories because the equations of motion of midges are not known; nevertheless, they still must obey basic kinematics such as smoothness of the trajectories, which is the only assumption underlying our tracking algorithm.

**Figure 4:**
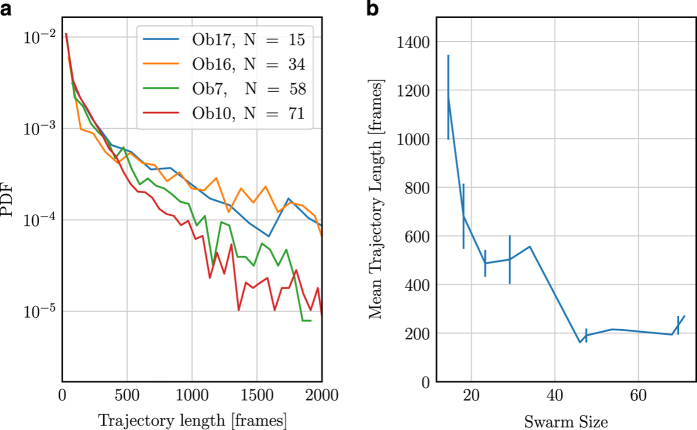
Statistics of track lengths for several swarms. (**a**) Probability density functions of track length for swarms containing between 15 and 71 individuals. The distribution of track lengths has a roughly exponential tail with a slight dependence of the decay factor on the swarm size. (**b**) The mean track length as a function of the swarm size. Larger swarms tend to have lower mean trajectory lengths.

The resulting distribution of trajectory lengths is shown in Fig. 4. These distributions have long, nearly exponential tails, implying that the ending of a trajectory is uncorrelated and random. There is a slight increase of the decay rate with swarm size, with larger swarms favoring shorter trajectories. This effect is likely due to a combination of factors, including a greater likelihood of visual occlusions on the cameras when more midges are flying and a higher chance of a midge leaving the field of the view of the cameras for larger swarm volumes. As a result, the mean trajectory length decreases somewhat with the total number of midges in the swarm. Note, however, that this does not mean that a smaller proportion of the midges are tracked. Rather, the conservative approach we take in reconstructing midge identity is more likely to result in broken trajectories.

**Figure 5:**
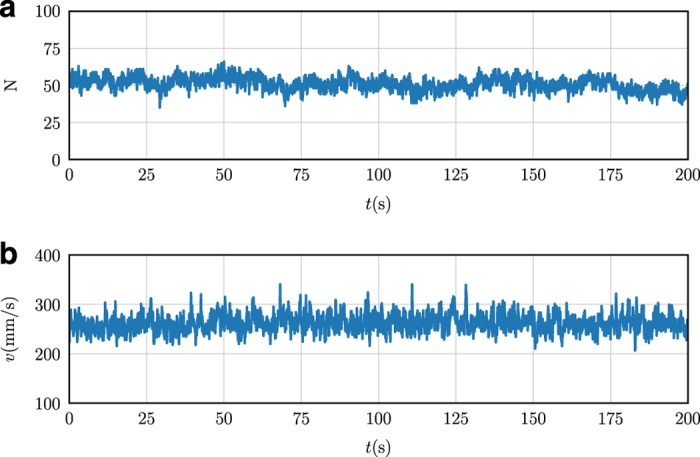
Time series of swarm properties for Ob14. (**a**) Time series of the number of midges in the swarm. The number of detected midges in the swarm remains roughly constant, with a mean value of 54 individuals and only small fluctuations. (**b**) Time series of the mean speed of midges from the same swarm. As expected, the speed is not a strong function of time and fluctuates about a common mean value. Neither of the time series show any indication of outliers produced by errors in the data processing.

**Figure 6:**
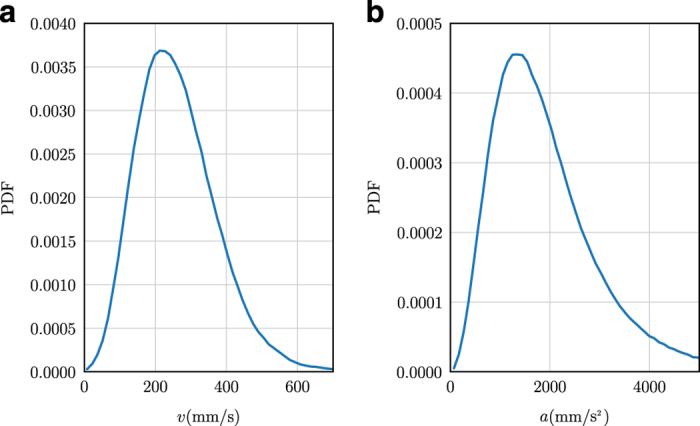
Probability density functions (PDFs) of midge kinematics for Ob14. (**a**) PDF of speeds of individual midges. The PDF is smooth with no unusual features. The tail of the PDF is well-resolved. (**b**) PDF of acceleration magnitudes of individual midges. As with the velocity PDF, the acceleration statistics are smooth and have no unexpected features.

As an a posteriori validation step of the quality of our data, we checked the kinematics of our midge trajectories for inconsistencies that might be the result of systematic errors. In Fig. 5, time series of the number of midges *N* and the mean speed *v* of midges from observation 14 are shown over the course of the entire measurement. Neither of these quantities show any suspicious outliers (that is, data points that are very far from the mean behaviour), and they agree with human observations of swarm size and the typical travel times of midges through the swarm volume. Similarly, the kinematic statistics of the midges, such as the speed and acceleration magnitude probability density functions shown in Fig. 6, are smooth and show no unexpected features.

## Usage Notes

While gravity is always directed in the negative *z* direction, neither the *x* and *y* direction nor the absolute position of the origin in space are fixed between different observations. The coordinate system for each observation was determined by the position and orientation of the calibration target, and the target was not placed at the exact same location and orientation for different measurements. However, the center of mass of the swarm defines a physical meaningful origin in each case, and we find that the swarms are azimuthally symmetric.

## Additional information

**How to cite this article**: Sinhuber, M. *et al.* The Subnational Human Development Database. *Sci. Data*. 6:190036 https://doi.org/10.1038/sdata.2019.36 (2019).

**Publisher’s note**: Springer Nature remains neutral with regard to jurisdictional claims in published maps and institutional affiliations.

## Supplementary Material



## Figures and Tables

**Table 1:** Overview of the individual swarming events in the dataset.

Observation ID	Dataset length [frames]	Mean swarm size	Mean track length [frames]
Ob1	11000	94	224
Ob2	14870	68	194
Ob3	15000	46	162
Ob4	15000	29	417
Ob5	15000	22	523
Ob6	20000	18	509
Ob7	10000	58	217
Ob8	19000	27	194
Ob9	10000	49	219
Ob10	15000	71	270
Ob11	20000	14	1028
Ob12	20000	19	335
Ob13	20000	27	525
Ob14	20000	54	212
Ob15	20000	20	549
Ob16	20000	34	559
Ob17	20000	15	1467
Ob18	20000	19	493
Ob19	30000	29	808
All swarms were observed at laboratory dusk apart from observations Ob6 and Ob11, which were captured during laboratory daytime.			

**Table 2:** Columns in a single swarm dataset.

Column	1	2	3	4	5	6	7	8	9	10	11
Data	*id*	*x*	*z*	*y*	*t*	*v*_*x*_	*v*_*z*_	*v*_*y*_	*a*_*x*_	*a*_*z*_	*a*_*y*_
